# Bioprospecting Marine Plankton

**DOI:** 10.3390/md11114594

**Published:** 2013-11-14

**Authors:** Heni Abida, Sandrine Ruchaud, Laurent Rios, Anne Humeau, Ian Probert, Colomban De Vargas, Stéphane Bach, Chris Bowler

**Affiliations:** 1 Environmental and Evolutionary Genomics Section, Institut de Biologie de l’Ecole Normale Supérieure (IBENS), Centre National de la Recherche Scientifique (CNRS) UMR8197 INSERM U1024, 46 rue d’Ulm, Paris CEDEX 05 75230, France; E-Mail: abida@biologie.ens.fr; 2 Kinase Inhibitor Specialized Screening facility (KISSf), Station Biologique de Roscoff, Centre National de la Recherche Scientifique (CNRS) USR 3151 (Protein Phosphorylation and Human Diseases), CS 90074, Roscoff CEDEX 29688, France; E-Mail: sruchaud@sb-roscoff.fr; 3 Greentech France, Biopôle Clermont Limagne, Saint Beauzire 63360, France; E-Mail: laurentrios@greentech.fr; 4 Soliance France, Centre de Biotechnologie Marine, Anse de Pors Gelin-Ile Grande, Plemeur-Bodou 22560, France; E-Mail: a.humeau@soliance.com; 5 Roscoff Culture Collection, Station Biologique de Roscoff, Centre National de la Recherche Scientifique (CNRS) USR 3151, Place Georges Teissier, CS 90074, Roscoff CEDEX 29688, France; E-Mail: probert@sb-roscoff.fr; 6 EPPO Laboratory, Station Biologique de Roscoff, Centre National de la Recherche Scientifique (CNRS) USR 3151, Place Georges Teissier, CS 90074, Roscoff CEDEX 29688, France; E-Mail: vargas@sb-roscoff.fr

**Keywords:** bioprospecting, high-throughput screening, plankton natural product, marine natural product, biodiversity, molecule discovery

## Abstract

The ocean dominates the surface of our planet and plays a major role in regulating the biosphere. For example, the microscopic photosynthetic organisms living within provide 50% of the oxygen we breathe, and much of our food and mineral resources are extracted from the ocean. In a time of ecological crisis and major changes in our society, it is essential to turn our attention towards the sea to find additional solutions for a sustainable future. Remarkably, while we are overexploiting many marine resources, particularly the fisheries, the planktonic compartment composed of zooplankton, phytoplankton, bacteria and viruses, represents 95% of marine biomass and yet the extent of its diversity remains largely unknown and underexploited. Consequently, the potential of plankton as a bioresource for humanity is largely untapped. Due to their diverse evolutionary backgrounds, planktonic organisms offer immense opportunities: new resources for medicine, cosmetics and food, renewable energy, and long-term solutions to mitigate climate change. Research programs aiming to exploit culture collections of marine micro-organisms as well as to prospect the huge resources of marine planktonic biodiversity in the oceans are now underway, and several bioactive extracts and purified compounds have already been identified. This review will survey and assess the current state-of-the-art and will propose methodologies to better exploit the potential of marine plankton for drug discovery and for dermocosmetics.

## 1. Introduction

As the world population expands and ages, we need to prepare to face new challenges if we hope to live longer and healthier lives while conserving our planet and its natural resources for future generations. Health care is one of the major concerns linked to increased life expectancy and the recent shift in lifestyles. On the one hand, the increase in genetic and life-style related diseases such as cardiovascular disease, ischemic stroke, diabetes, chronic respiratory disease, and some types of cancers are on the rise [1] and on the other hand, despite remarkable progress, infectious diseases persist as being the leading causes of mortality in the world. Indeed, even with a supposedly large arsenal of therapeutic methods, infections continue to be a major health concern all over the world—including developed countries [2]. Unfortunately, the discovery of new drugs has not been following the curve dictated by the emergence of drug-resistant bacteria: since the golden era of antibiotics in the 1940s, the discovery of novel antibacterial agents has been slowing down, and today the R&D pipeline for such molecules has practically run dry [3]. Unless the rise in antibiotic resistance can be reversed, we can expect to see a substantial rise in incurable infection and fatality in all parts of the world [4,5].

When it comes to life-style diseases, although our therapeutic arsenal is very limited, some simple adjustments to our nutrition can considerably reduce the risk of such conditions [6], but feeding over seven billion people with healthy and nutritious food is no easy task. In addition to having more and more people to feed, current climate changes are causing reduced yields of some of our most common crops on a global scale [7,8], which may have particularly dire effects in developing regions of the world. In fact, it is not just the crops but entire ecosystems that may be remodeled as we continue to burn fossil fuel and release carbon in the atmosphere at a rate one million times faster than it takes the planet to sequester it [9]. Our energy consumption has gone from 8.5 million to over 13 million kilotons of oil equivalents in the past twenty years [10], and yet we remain almost entirely reliant on fossil fuels.

The issues of health, nutrition and energy are all becoming increasingly substantial, but are also strongly intertwined: we must bolster our therapeutic options in spite of the constant race against drug-resistant strains, find new and better ways to feed an increasing population while keeping a healthy diet, and reduce our carbon emissions drastically despite our heavy reliance on oil. Addressing one issue without exacerbating another has proven to be quite a challenge in the past decade. One example is that the 250-fold increase in nitrogen production over the past century (principally driven by its use as fertilizer in agriculture) compares with only a three-fold increase in the human population over the same time period. However, the excess nitrogen fertilizer in soil and run-off water feeds certain bacteria which release nitrous oxide into the atmosphere, the third most potent human contributor to global warming today [11].

We believe that novel and less disruptive solutions could be found through bioprospecting the oceans, in particular drawing on the arsenals of molecules, enzymes and genes found in largely unexplored groups of microscopic marine organisms. Recent advances in DNA technologies and heightened awareness of environmental issues, such as global warming, have come together to potentiate the science of marine microbiology [12]. Many international research initiatives have emerged in the past few decades, such as the US-EU marine genomics taskforce on biotechnology research, the *Tara*-Oceans and Malaspina expedition, the MicroB3 Project, the Global Ocean Sampling expedition, Ocean Sampling Day, and the PharmaSea project. Furthermore, our improved understanding of microbial communities, coupled with new technologies for sampling the ocean and for bioactive screening, has led to the identification of a range of molecules, genes, and strains of interest. In this review, we will discuss how bioprospecting of these organisms can provide us with tools to address key issues facing humanity in the form of novel bioactive molecules and novel high-producing strains and describe screening methodologies that have brought such molecules and/or strains to light.

## 2. The Plankton as a Target for Bioprospecting

About 3.5 billion years ago the ocean was probably the birthplace of life, and today marine microbes number in the millions in every liter of seawater. They represent more than 95% of marine biomass and are present in many different environments, e.g., in the water column, in ocean sediments, or associated with other organisms. Many of them drift with the currents together with other microscopic organisms such as zooplankton and are collectively referred to as plankton, from the Greek word “planktos” meaning “drifter”. Planktonic organisms are found in all marine environments, including extreme conditions, and are extremely diverse in taxonomic groups (with representatives from all kingdoms of life), trophic groups and sizes (Table 1). Representatives include viruses, bacteria, the photosynthetic phytoplankton, and a wide range of larger zooplankton that graze on the smaller organisms. The abundance of different plankton varies according to size, with viruses present typically at up to 10 billion particles/L, bacteria at up to one billion cells/L, phytoplankton at up to 10 million cells/L, and zooplankton at up to 1000 organisms/L. Marine planktonic ecosystems are highly dynamic environments subject to a wide range of external forces. Some organisms, referred to as holoplankton, are constitutively planktonic whereas others (known as meroplankton) are part of this community only during a specific phase of their life cycle, usually the larval stage. It is important not to overlook these latter organisms in bioprospecting because they may represent novel sources of molecules (e.g., used in defense mechanisms) that are not found in their mature counterparts. Such metabolites have been reported in larvae from *Luffariella variabilis* [13], asteroid eggs [14], ascidian larvae [15], polychaetes [16], and bryozoan larvae [17], none of which were detected in their mature—non-planktonic—counterparts.

**Table 1:** The different size classes of plankton, together with representatives of taxa present in each. Colouring is as follows: 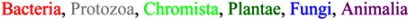.

Plankton breakdown by size	Examples of diversity by taxonomy
Megaplankton Over 20 mm	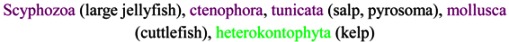
Macroplankton From 2 mm to 20 mm	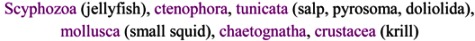
Mesoplankton From 0.2 mm to 2 mm	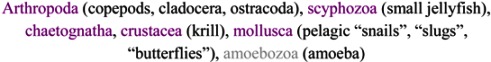
Microplankton From 20 µm to 200 µm	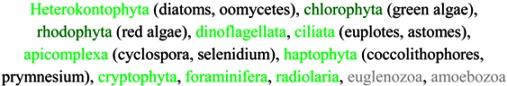
Nanoplankton From 2 µm to 20 µm	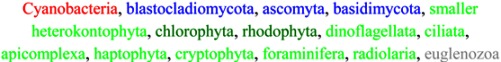
Picoplankton From 0.2 µm to 2 µm	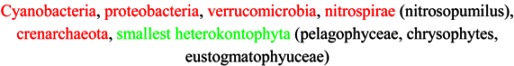
Femtoplankton	Viruses

Plankton are also often distinguished based on their mode of nutrition: autotrophic planktonic organisms use sunlight to drive photosynthesis and are referred to as phytoplankton, while the planktonic organisms that graze on them are called zooplankton and include both single celled protists such as ciliates and multicellular organisms such as salps and copepods. However, the line between phytoplankton and zooplankton can be blurred as some organisms are capable of both autotrophy and heterotrophy simultaneously, or alternate between the two depending on their environment. Due to their mixotrophic metabolism, their potential for exploitation may be missed because some functions are only transiently expressed.

Bioprospecting can involve the collection of organisms and subsequent screening for a specific molecule or activity of interest. An alternative to prospecting directly for bioactives is to search for DNA sequences encoding activities of interest, either from single organisms or by mining metagenomic sequencing data derived from whole plankton communities collected from the water column [18]. Such approaches can help bypass a number of steps required in molecule screening. Genes encoding enzymes that perform particular chemical reactions of interest to industry are well known examples (see later). Additionally, genes from plankton can be transferred to other organisms using transgenic approaches. As a case in point, one approach to reducing nitrogen input in agriculture could be to improve nitrogen use efficiency in crop plants by transferring genes encoding nitrogen transporters from phytoplankton, because these organisms are adapted to environments in which nutrients such as nitrogen are extremely scarce. Using genes from such organisms for engineering crops could perhaps yield impressive results. In addition to identifying specific molecules and genes in planktonic ecosystems, individual species and strains could allow us to produce molecules of interest that we are already familiar with but can not yet produce cost-efficiently or sustainably. Some marine microorganisms are fast biomass producers and contribute significantly to global primary production [19], which makes them strong candidates to produce molecules of interest at a low cost and with little energy input. In fact phytoplankton have been used for a long time as food for humans [20] or as feedstock in aquaculture [21]. Besides being rich sources of proteins, some species like the diatom *Phaeodactylum tricornutum* are very efficient producers of other value-added compounds such as polyunsaturated fatty acids (PUFAs), carotenoids, phycobiliproteins, polysaccharides, vitamins, and sterols [22]. Some staple products in scientific research are extracted from plankton as well, such as agar which is produced by *Gracilana edulis*. Additionally, some phytoplankton such as *Nannochloropsis gaditana* and *P*. *tricornutum* are proficient at producing triacylglycerols (TAGs) which can be processed into a variety of biodiesel blends. While there are still many obstacles that must be overcome before microalgal biofuel production can become cost-efficient, the field is developing very rapidly and novel TAG-producing species and strains are constantly being discovered, a recent example being *Fistulifera* sp. JPCC DA0580 [23,24].

## 3. Bioactives from Marine Sources

**Figure 1:**
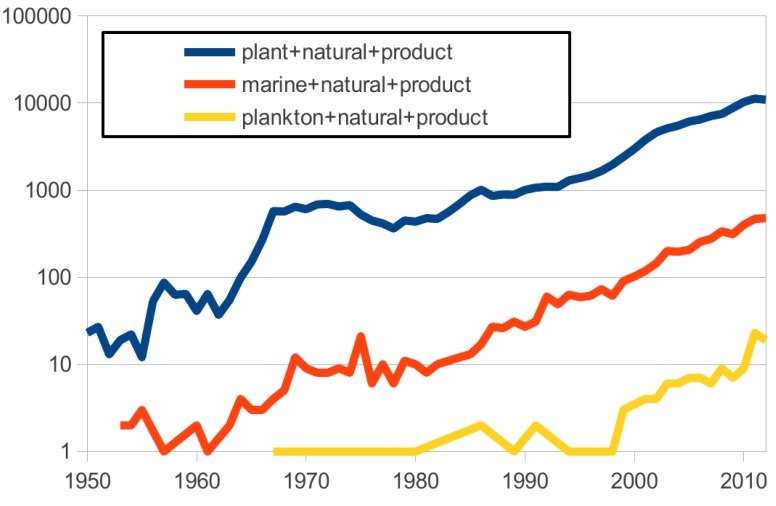
Number of articles per keyword query by year (analysis done on 26 August 2013 using PubMed.gov).

With many examples already (1003 new compounds described in 2010, more than 10,000 since the 1960s [25]), the number of articles describing “marine natural products” has increased in the last 50 years but remains significantly lower than those describing “plant natural products”. With a similar trend, articles describing “plankton natural products” are increasing as well, but there is still a long way to go and this trend only began in the late 1990’s (Figure 1). Conversely the number of companies active in the development of natural products from plankton has steadily increased. Amongst these, Greentech and Soliance are specific examples of companies with products derived from marine plankton (see later). Planktonic organisms, notably microalgae, generate a wide range of primary and secondary metabolites of commercial interest. The most well known examples are from chlorophycaea: β-carotene from *Dunaliella tertiolecta*, an industry currently estimated at $260 million and astaxanthin from *Hematococcus pluvialis*, which generated $200 million in 2012. Of course, one species can be efficient at producing more than one high-value compound simultaneously: *D*. *tertiolecta* is also known to produce a pigment, violaxanthin, that exerts a potent antiproliferative activity on MCF-7 breast cancer cells [26].

Mining for novel molecules in planktonic communities has already delivered a wide range of additional molecules of biomedical interest, including cyclopeptides from cyanobacteria which could be novel cancer drugs [27], domoic acid from red algae and diatoms for its anthelmintic activity [28], fucoxanthin from brown algae as a possible treatment for cancer [29] or to combat diabetes [30,31], cephalosporin P from fungi as an antibacterial agent [32], diterpenoid diacylglycerol from planktonic molluscs as protein kinase C activators [33] and phlorotannins from kelp [34] and many more are in (pre-)clinical trial phases. Organisms adapted to extreme conditions (temperature, pressure, pH, salinity, toxicity, *etc*.) seem particularly interesting for finding molecules that would not normally be encountered in the most common environments, whether they be primary or secondary metabolites. For example, extremophilic bacteria have been sampled extensively in such environments, which has led to the identification of a range of novel molecules, most notably the DNA polymerases now exploited for the polymerase chain reaction [35] a workhorse technique in molecular biology.

**Figure 2:**
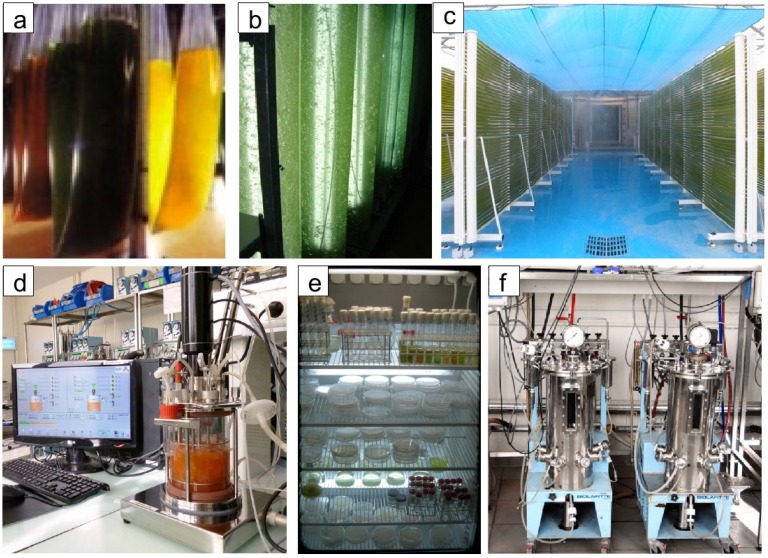
Culturing methods for microalgae developed at Greentech. (**a**) “Bag” culture system, very efficient at producing biomass at a low cost in batches. From left to right: Red algae (two bags), Green algae (two bags), Green algae in optimal conditions for β-carotene production, hence the yellow color (two bags); (**b**) “Annula” photobioreactor where the artificial light source is inside the culture. This system is optimal for controlling culture conditions and mixing, which generally allows for the highest final biomass yield in batch cultures; (**c**) 2500 L “Tubular” photobioreactor. This system is best for continuous culture conditions while maintaining the bulk of the culture in exponential growth phase; (**d**) Bench photobioreactor used by Greentech for culture condition optimization; (**e**) Samples from a culture collection in liquid or solid media; (**f**) “Fermentor” culture system for heterotrophic microalgae growth.

The biotechnology company Greentech (Saint-Beauzire, France) uses natural raw materials (plants, seaweeds, microalgae and microorganisms) to obtain natural molecules of interest and to develop them into innovative bioactive molecules. Greentech aims at the development of technologies using algae (mainly microalgae) as raw materials or as biological tools to produce products or services. With this knowledge, Greentech has isolated a variety of microalgal strains and has optimized culture conditions—photoautotrophic as well as heterotrophic—in closed systems (Figure 2) in order to produce and study their metabolites. Indeed, many of these metabolites are produced transiently by microalgae as a response to stress which has led to the development of a technology denoted metabolic induction. Microalgae are efficient at adapting to rapid environmental changes and therefore the metabolic induction concept lies in managing and modifying culture parameters during the final production step in bioreactors which triggers physiological adjustments that induce the over-expression or accumulation of the targeted metabolites. By using metabolic induction, metabolite production can allow efficient harvest of value-added components without using genetically modified organisms. Such technologies have allowed industry-scale production of:

- Nutrients and oligoelements commercialized for human nutrition or aquaculture: polyunsaturated fatty acids (PUFAs), including eicosapentaenoic acid (EPA) and docosahexaenoic acid (DHA), carotenoids, and modified amino acids such as selenomethionine.
- Fluorescent pigments commercialized as fluorescent markers for biological or medical diagnosis as a non-polluting—yet equally efficient—alternative to radioimmunological tracers, e.g., C-/R-phycocyanin, allophycocyanin, and C-/R-phycoerythrin.

Greentech has also developed active ingredients for cosmetics such as SILIDINE^®^ from the microalga *Porphyridium cruentum*. SILIDINE^®^ is a blend of oligosaccharides and oligoelements that induce the synthesis of endothelin-1 in fibroblasts and endothelial cells when applied topically. The binding of endothelin-1 to surface receptors (ET_A_ and ET_B2_ receptors) of vascular smooth muscle cells induces a signal that spreads from the outside to the inside of the plasma membrane and activates several effectors that initiate release of the second messenger Ca^2+^, formation of the complex Ca^2+^-calmodulin-myosin light chain kinase, phosphorylation of the myosin light chain and finally constriction of the microvasculature. This bioactive blend can be used for vascular tonicity enhancement to correct redness (rosacea, flushing, dark circles) and heavy legs syndrome.

As another proof-of-concept from industry, the Soliance Company (Pomacle and Pleumeur-Bodou, France) has developed an innovative active ingredient, Megassane, from the cultivated marine microalgae *P*. *tricornutum* which is reputed as being rich in eicosapentaenoic acid (EPA) and docosahexaenoic acid (DHA), two essential polyunsaturated fatty acids (PUFAs). The initial objective of Soliance’s screen was to isolate an anti-aging agent by screening for molecules that target proteasome activity. The proteasome is a proteolytic system responsible for the degradation of oxidized proteins (and was the object of the 2004 Nobel Prize in Chemistry awarded to Aaron Ciechanover, Avram Hershko and Irwin Rose). This enzymatic complex is in charge of “housekeeping” cellular cleansing through the degradation of damaged proteins. It is now well established that the proteasome is responsible for the degradation of most proteins and that impairment of proteasome function is a hallmark of cellular aging, most notably in photoaging (exposure of human skin to UV irradiation) and chronological aging [36].

Because fatty acids seem to have an influence on proteasome activation, the effects of oleic, linoleic and linolenic acids were studied by Soliance. Lipid extracts from *P*. *tricornutum* were tested on healthy human keratinocytes (NHK) and effects on the three proteasome proteolytic activities were analyzed: LLVY-amc, chymotrypsin-like activity; LLE-na, post-glutamic-like activity; LSTR-amx, trypsin-like activity. Megassane, thanks to its unique composition in fatty acids, was found to stimulate all these enzymatic activities and thus to activate the proteasome. Through this activity, Megassane helps the epidermis to eliminate oxidized proteins, as verified by quantification of oxidized proteins in NHK cells.

## 4. Bioprospecting in Culture Collections

The biodiversity of marine plankton is immense [37] and consequently so is the potential for biotechnological exploitation. While *ex situ* laboratory cultivation is not a prerequisite for bioprospecting, it tends to greatly facilitate it. For marine plankton, many microalgae and prokaryotes have been cultured, but there has been relatively little success for metazoan zooplankton, heterotrophic protists or viruses, perhaps because they have very specific requirements for vitamins, cofactors or micronutrients, or that they have very elaborate symbiotic or host-pathogen relationships that cannot currently be mimicked in controlled laboratory conditions. Even though a relatively wide range of cultures of microalgae and bacteria are available, the vast majority of applied research using cultivated resources has focused on a very limited number of model species/strains. There are a number of probable reasons for this, one of the main ones being a general lack of knowledge about the extent of the diversity of these organisms and of the culture conditions required to grow them.

Marine microalgae and bacteria were first cultured in the latter half of the 19th century [38]. Many of the methods and basic culture medium concepts that are used today were developed in the late 1800s and early 1900s. In line with the recent advances in culturing technologies (Figure 2) driven largely by interest in algae for biofuel production, several new culture media have been developed in recent years, and some older media have been improved. The use of flow cytometers for automated single cell isolation and the development of cryopreservation techniques for long-term maintenance of microorganism cultures are two important advances that have been made in recent times, leading to rapidly increasing numbers of culture isolates. Most cultures are isolated by research teams with specific uses in mind, but generally these laboratories do not have the means or the vocation to maintain these resources over the long-term.

In light of the generally accepted view that cultures are a very important community resource that are of key interest in diverse fundamental and applied research contexts, a number of dedicated structures for long-term maintenance and distribution of culture strains have been set up in the last 30 years. The main open-access service culture collections of marine microalgae (currently holding between 1000 and 3000 strains) are: the National Center for Marine Algae and Microbiota (NCMA—formerly the CCMP) [39] in the USA, the Culture Collection of Algae and Protozoa (CCAP) [40] in the UK, the Roscoff Culture Collection (RCC) [41] in France, the National Institute for Environmental Studies microbial culture collection (NIES) [42] in Japan, and the Commonwealth Scientific and Industrial Research Organisation culture collection (CSIRO) [43] in Australia. A number of large collections of marine prokaryote cultures also exist around the world, but these have a greater tendency to be developed and maintained by private research companies and consequently are not always open-access.

Culture collections represent a resource with outstanding potential for bioprospecting. The rationale for screening existing cultures lies in: (I) The potential to provide a global overview of metabolic capacities in different lineages that allows identification of groups meriting more detailed investigation (including, if necessary, targeted isolation of species not previously cultured); and (II) The fact that once a compound of interest has been identified in a given strain, metabolic variability in closely related strains (intraspecific/intrageneric diversity) can be quantified in order to attempt to identify strains capable of producing higher amounts of the compound in question. In this latter context, culture collections often maintain multiple strains (tens or sometimes even hundreds) of certain species isolated from different locations, seasons or depths. Economic constraints mean that most envisaged biotechnological applications would require strains that grow rapidly and easily, therefore being amenable to mass culture on an industrial scale. This is probably not the case for many (or even most) of the strains maintained in culture collections, but again the range of strains available should allow compromises to be found between capacity for growth and capacity to produce a compound of interest.

For microalgae, nearly all of the known main lineages have representatives in culture and it has been estimated that between 10% and 40% of described species in three of the main ecologically important marine lineages (diatoms, dinoflagellates, haptophytes) have been successfully cultured [44]. For heterotrophic eukaryotes and metazoan zooplankton the success rate is much lower and there are several main lineages that are yet to be maintained in long-term (multi-generational) culture, such as foraminifera and radiolaria from the Rhizaria supergroup. There is therefore considerable scope for increasing the range of cultures available for targeted screening programs. Recent international collaborative initiatives to specifically address this issue include the European FP7 project Marine Microorganisms: Cultivation Methods for Improving their Biotechnological Applications (MaCuMBA) [45].

For certain model microalgal species for which large-scale genomic data is available, genetic resources (e.g., insertion/deletion mutant collections) exist or are currently being generated. The best-known case of genetic transformation in microalgae is for the freshwater chlorophyte *Chlamydomonas rheinhardtii* [46], but techniques have also been successfully applied to marine microalgae such as the diatom *P*. *tricornutum* [47] and the prasinophyte *Ostreococcus tauri* [48]. Production of genetic resources is a highly specialized and extremely time-consuming activity that is typically undertaken by research teams in public research institutions. Genetic resources for marine microalgae should start to become publicly available in the very near future, potentially via service culture collections, and this will represent an important step for future development of biotechnological applications such as the production of bioactive compounds.

To improve the productivity of a molecule of interest from a strain in culture, a variety of approaches can be followed, from increasing cell doubling time, stationary phase biomass yield, or quantity of the desired compounds in each cell. To achieve such results substantial fundamental knowledge must be acquired first, such as a better understanding of cell-cycle, photosynthetic efficiency and nutrient uptake, or the detailed elucidation of a biosynthetic pathway of interest in order to overexpress enzymes involved in production or positive regulation, or to knock-down/out genes encoding negative regulators or enzymes that break down target molecules.

## 5. Bioprospecting in the Ocean

The bioprospecting approach is not new, as evidenced by the fact that more than 10,000 molecules have already been reported from marine sources, although very few of them have been put on the market [49]. Marine bioprospecting has tended to target macro-organisms such as corals and sponges because of their evolutionary diversity and because they have been assumed to be rich in defense molecules with efficient potencies adapted for dilute marine environments, but significant efforts have also targeted the deep ocean, particularly around hydrothermal vents because of the largely untapped biodiversity and unknown adaptations present in such extreme conditions [50]. Companies that have had a particularly high profile in the field of marine bioprospecting in recent decades include Diversa and New England Biolabs. However, perhaps due to their dynamic heterogeneous nature, or the fact that they are poorly understood, plankton ecosystems have received only scant notice from marine bioprospectors. In particular marine protists have been given very little attention despite their incredible diversity in planktonic communities, being estimated at between 70,000 and 300,000 species [51,52]. This lack of attention can be attributed to the fact that they are present in lower numbers than bacteria, making it more difficult to collect sufficient biomass. As highlighted in the previous section, an additional bottleneck is due to the difficulties of getting them into culture, although culturing techniques are evolving quickly (Figure 2).

Notwithstanding, marine protists are now receiving some attention thanks to global sampling expeditions such as *Tara*-Oceans [53,54] and Malaspina [55]. Both of these research expeditions involved around-the-world expeditions and sampling in underexplored planktonic territories, in the case of *Tara*-Oceans in many open ocean environments and in the case of Malaspina in the twilight zone between the surface euphotic zone and the deep ocean known as the mesopelagic. Both of these expeditions have brought back thousands of samples that are now being analyzed by microscopy and by DNA sequencing. Although the primary objective of these projects is to learn more about the fundamentals of marine plankton biodiversity and the functioning of their microscopic ecosystems, it is obvious that these efforts may lead to the discovery of genes encoding products and processes that can be exploited commercially.

Current interest in bioprospecting in the oceans has been further potentiated by the integration of high-throughput DNA sequencing methods to evaluate planktonic diversity and their gene repertoires. Such genomic data can provide a useful starting point to identify new enzymes involved in the biosynthesis of secondary metabolites. As a case in point, modular enzymatic assembly lines that generate molecules such as polyketides, non-ribosomal peptides, and their hybrids have been of particular interest because they can easily be identified in metagenomic and metatranscriptomic screens of natural samples [56].

The production of bioactive metabolites by plankton species generally occurs during competition for resources, suggesting that it may be possible to identify oceanic regions that can be specifically targeted for bioprospecting, e.g., oligotrophic areas in the open ocean. For example, triacylglycerides are typically induced during nutrient starvation because they constitute important energy storage compounds that prolong cell survival in unfavorable conditions [57]. Conversely, oxylipin production by diatoms has been proposed to be induced as a defense mechanism against their principal predators, crustacean copepods [58], and to constitute an effective system for allelopathic signaling between diatom cells, a cellular cross talk mechanism thought to be essential to such marine communities [59]. The cytotoxic effects of some of these diatom-derived aldehydes were also found in organisms belonging to different phyla ranging from bacteria to marine invertebrates [60]. Furthermore, some molecules may even be generated as the result of cooperative chemistry between host and microbial photosymbionts and/or bacterial symbionts. For example, it was demonstrated in *Dysidea avara* that the level of metabolites produced was dependent on the nature of their neighbors [61]. It is therefore crucial to consider a variety of planktonic organisms for bioprospecting, rather than narrow the search down to a specific clade or size fraction.

Because marine genetic resources (MGRs) have the potential to sustainably deliver considerable wealth and business opportunities to both local and multinational economies, the access and fair sharing of benefits (ABS) arising from the exploitation (for commercial purposes or for research) of MGRs is essential. Plankton are a particularly important case in point because they drift in ocean currents independently of national territories. Remarkably, more than two thirds of the ocean’s surface area is beyond national jurisdiction, being governed by the United Nations Convention on the Law of the Sea (UNCLOS), which essentially means that it is not governed by anyone. An Ad Hoc working group entitled BBNJ (marine biodiversity in areas beyond national jurisdiction) is aiming to resolve issues relating to the conservation and sustainable use of marine bioresources from the high seas by 2015 and to implement a legal framework for managing biodiversity. To facilitate dialogue among the relevant stakeholders (governments, industry, research), the Mediterranean Science Commission (CIESM) has drafted a Charter on ABS that would apply to parties engaging in the collection and exploitation of MGRs. It is built upon nine core values to favor marine research and development, while preventing abuses of the ocean global commons [62]. The Paris Appeal for the High Seas also aims to establish a legal framework for the high seas to be managed as a shared common heritage for all humanity [63]. In our opinion it is essential to convince all countries as well as the United Nations to allow free sampling of ocean ecosystems to scientists investigating the structure of living systems in the oceans, as well as to develop international agreements that govern the commercial use of scientific data derived from MGRs that takes equitable account of the interests of basic and applied research. Equally important is the need for fair play in ABS issues, to ensure that scientific capacity building is transmitted from countries such as the USA and EU member states to less developed southern countries.

## 6. High-Throughput Screening of Planktonic Resources

**Figure 3:**
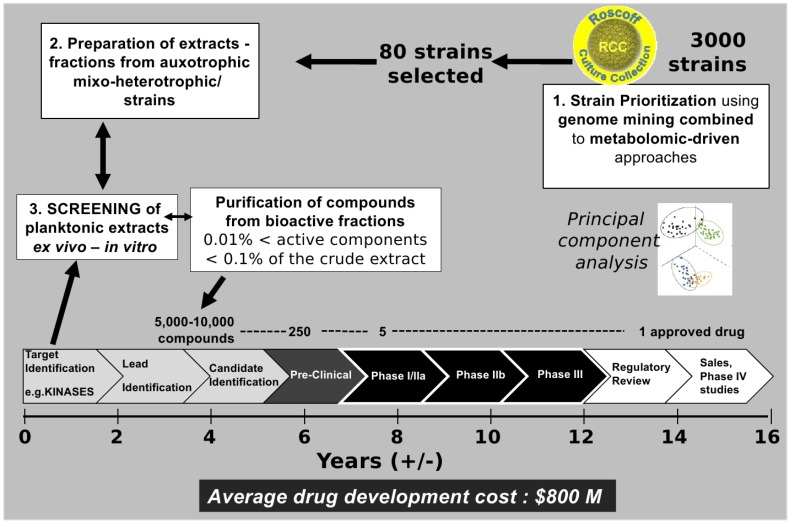
From cultivable plankton strains to drugs on the market: A long-term adventure.

The diversity of planktonic species (Table 1), from megaplankton to marine viruses, suggests an immense chemical diversity. A major obstacle when bioprospecting for natural products is that many resources are wasted in re-purifying and re-characterizing non-novel compounds only to discover—after time and money have been invested in sampling, cultivation, extraction, fractionation, purification and structural elucidation—that these compounds are already among the 246,000 natural compounds that have already been described [64]. To avoid the re-isolation of already described bioactive compounds and to potentiate the screening efforts, it is crucial to identify these molecules in crude marine extracts as early as possible. This strategy is called “dereplication” and involves various separation and analytical techniques (e.g., high-resolution mass spectrometry [64]). The number of studies using dereplication methods is constantly growing. Various strategies can simplify the dereplication process: one of them is to optimize the choice of marine strains to extract based on genomic and/or metabolic mining (Figure 3). For example, potential metabolite producers have been identified in collections of cultivable plankton strains by screening for specific gene encoding enzymes known to be involved in the biosynthesis of secondary metabolites of interest [65,66]. The modular enzymatic assembly lines, polyketide (PK), nonribosomal peptides (NRP) and hybrid NRP-PK are now very well described and can be targeted [56]. Besides strain prioritization, genomic/metabolomic approaches can be applied to further optimize growth conditions to increase specific metabolite production (e.g., using LC/MS-PCA-based methods [67]). The major challenge is thus to obtain a gain in originality in terms of chemical diversity of the secondary metabolites, although using a variety of culture conditions may be one way to improve this diversity. Some methods such as “OSMAC”, for One-Strain-Many-Compounds, suggest modification of conditions such as medium composition, temperature, aeration, pH, and enzyme inhibitors [68].

Various extraction techniques can be used to obtain sufficient amount of crude extract to complete the isolation of active molecules and achieve their structural elucidation but this information is readily found in the literature and will not be discussed here (for fungi: [69], for microalgae: [70]). In the process of natural product drug discovery, bioassay results guide the purification processes towards increasingly small fractions. Extracts from selected strains can be analyzed using various cell-based and biochemical assays. One example concerns the screening for molecules that target human disease-related protein kinases (PKs). PKs are currently among the most promising targets used by big pharmaceutical companies for the characterization of new active ingredients. Robotic-assisted methods are now available to perform a fully-automated analysis of potential PK inhibitory activity in natural extracts or arrays of purified chemical compounds [67]. As a case in point, SB-Roscoff has developed a Kinase Inhibitor Specialized Screening facility (KISSf, Roscoff, France) using a panel of selected disease-related PKs in order to evaluate the therapeutic properties of either marine natural extracts or purified natural compounds.

Fields of application depend on the kinases being targeted. PKs involved in the control of cell division (notably cyclin-dependent kinases, CDKs) or more precisely in mitosis (Aurora and Polo-like kinases) may be of interest for cancer treatment. To target similar kinases essential for the cycle of parasites such as those responsible for leishmaniasis or malaria, parallel screenings may be carried out in order to compare the specific inhibition of parasite-derived kinases *vs*. human kinases in order to develop new therapeutic methods. The potency to inhibit cell growth can be combined with screening methods on PKs to optimize the “bio-guided” fractionation and isolation of active planktonic compounds [26]. With this kind of automation, and our increasing knowledge in cell culturing, an impressive screening potential is made available through planktonic micro-organism screening in which cell fractions can be cultured in a variety of conditions and then screened for the desired active agent at a rate that is unmatched. Essentially, the successful screening methods used for prokaryotes and yeast from land environments can be expanded to an even more diverse environment. The screening of marine extracts in the general context of drug development is depicted in Figure 3. Drug development defines the process of bringing a new drug to the market once a candidate compound has been identified through the process of drug discovery including notably the screening phase. Drug development includes pre-clinical research on microorganisms/animals and clinical trials on humans and usually includes the obligatory step of obtaining regulatory approval to market the drug. Figure 3 highlights the complexity of the process. Indeed, it is estimated that the success rate for the development of a new drug is 0.01% [71].

## 7. Conclusions

From this brief overview in which we have summarized current state-of-the-art and prospects for discovery of new bioresources from marine planktonic organisms, it is evident that several success stories have already emerged and that interest in the field is likely to grow way beyond what has been seen in previous years. Bioprospecting the immense planktonic biodiversity could lead to new ways to address a number of today’s global issues. Using phytoplankton as a feedstock for biofuels would have a drastically lower impact on the planet’s carbon cycle than using fossil fuels, could provide us with sustainable liquid energies such as biodiesel, and perhaps even hydrogen some day. Planktonic organisms could also help us produce large amounts of nutraceutical and pharmaceutical components to help the increasingly aging population by preventing or treating lifestyle-related diseases. Additionally, mining for new bioactive molecules in this virtually unchartered ecosystem could yield a variety of pharmaceutical components that would feed a nearly dry antibiotic pipeline and help fight against the inevitable emergence of multi-drug resistant microbes.

Furthermore, this is also a significant moment of converging influences for the field. On the one hand, new technologies for DNA sequencing are driving organism and gene discovery from environmental samples at an unprecedented rate, as well as for the high-throughput screening for identification of compounds and/or extracts with bioactivities of commercial interest. On the other hand, planetary-scale research expeditions are harvesting samples from which new leads for drug discovery can arise. Notwithstanding, our ability to culture marine micro-organisms has not advanced to the same degree, and a legal framework for granting access and sharing of benefits from marine bioprospecting is yet to emerge. The immediate future holds great promise, particularly if these obstacles can be breached, and the best path forward will be through an enhanced two-way collaboration between researchers in the public and private sectors, as well as a more active exchange between scientists and legal authorities charged with establishing a workable basis for an equitable bioprospecting of the ocean.
